# Aging, frailty, and design of built environments

**DOI:** 10.1186/s40101-021-00274-w

**Published:** 2022-01-03

**Authors:** Douglas E. Crews

**Affiliations:** Department of Anthropology and School of Public Health, The Ohio State University, 4034 Smith Laboratory, 174 W. 18th Avenue, Columbus, OH 43210-1106 USA

**Keywords:** Accessibility, Accommodating, Aging, Design, Frailty, Housing, Mobility, Seniors

## Abstract

Before developing agriculture, herding or metallurgy, humans occupied most of the world. Multiple socioculturally-based responses supported their migration, including building shelters and constructing niches to limit environmental stressors. Sheltered settings provided social support and security during stressful times, along with opportunities for injured, aging, and frail members to survive. Modern built environments are designed for similar purposes, to support human growth, development, reproduction, and maintenance. However, extended survival in modern settings has costs. With age, muscle (sarcopenia) and bone loss (osteopenia, osteoporosis), along with somatic, physiological, and sensory dysfunction, reduce our physical capabilities, increase our frailty, and impede our abilities to interface with built and natural environments and manufactured artifacts. Thereby, increasing our dependence on built environments to maintain autonomy and quality of life.

What follows is a conceptual review of how frailty may limit seniors within modern built environments. It suggests age-related frailty among seniors provides specific data for those designing environments for accessibility to all users. It is based in human ecological theory, and physiological and gerontological research showing senescent alterations, including losses of muscle, bone, and sensory perceptions, produce a frail phenotype with increasing age limiting our mobility, activity, use of space, and physical abilities. As an individual phenotype, frailty leads to age-related physical and performance declines. As a physiological assessment, frailty indices amalgamate individual measures of functional abilities into a single score. Such frailty indices increase with age and differ betwixt individuals and across groups. To design built environments that improve access, usability, and safety for aging and frail citizens, today’s seniors provide living samples and evidence for determining their future abilities, limitations, and design needs. Designing built environments to accommodate and improve the quality of human-environment interactions for frail seniors will improve usability and accessibility for most user groups.

## Background

Human habitation of Earth’s many environments prior to developing agriculture or metallurgy depended on sociocultural and biocultural systems organized to build livable microenvironments wherever they roamed [1, 2]. Along with providing microenvironments wherein adults could provision altricial offspring over an extended period, this strategy supported communal food procurement and defense [1–4]. Thereby, also supporting humankind’s evolving life history (LH: timing of gestation, infancy, childhood, attainment of adult form, reproductive effort, survival), including dependent young and biocultural reproduction [2, 4]. Modern human offspring require parental investment over approximately two decades before they are sufficiently mature to not only produce offspring, but also sufficiently skilled socially to nurture and fledge them [2, 4–6]. Over human evolution, fully dependent altricial offspring became our species’ hallmark, along with culture, language, sociality, tool use, and built environments. These contributed to reduced stressor exposures and aided humans in retaining sufficient physiological capacity to survive decades beyond their reproductive prime.

This shared adaptive suite in conjunction with humankind’s physiological adaptability produced an array of modern regionally successful phenotypes, inhabiting multiple environments, all maintaining sufficient physiological capacity to survive 75+ years (Table 1) [1, 2, 6, 7]. High survival to late life, ages 75+ years, worldwide suggests emergence of a post-reproductive human LH period. One characterized by investing remaining somatic capacities into somatic maintenance and survival. As survival has improved, ubiquitous age-related somatic declines have been observed: physiological dysregulation, sensory losses, sarcopenia, osteoporosis, chronic degenerative conditions, and non-communicable diseases (NCD); along with detrimental alterations in our genome, microbiota, cells, tissues, and organs. Collectively, stressors, whether intrinsic or extrinsic, negatively alter neurological, physiological, and somatic function contributing to senescent change and dysfunction as we survive past our prime reproductive years [8–11]. Here, I explore how frailty, a remodeling of somatic structures secondary to muscle and bone loss characterized by reduce physical and sensory abilities, provides an evidentiary base likely to aid those designing and redesigning built living spaces to improve accessibility by increasingly frail seniors, while also accommodating their limitations.

**Table 1:** Percent born surviving to selected ages (%) and percent in population (%p) at specific ages 70 and 85 years plus, and life expectancy at age 75 years in selected nations

	Age 70						Age 85			
Nation	Men			Women			Men		Women	
	%	%p	e_x_	%	%p	e_x_	%	%p	%	%p
Japan	75	(9.0)	13.5y	88	(13.5)	17.7y	29	(1.3)	59	(2.3)
UK	70	(8.9)	11.9y	81	(13.5)	14.7y	21	(1.0)	39	(2.8)
USA	65	(7.4)	12.8y	79	(10.8)	15.5y	24	(0.9)	42	(2.1)
Sweden	75	(10.7)	12.8y	85	(15.3)	15.9y	27	(1.4)	47	(3.1)
Ecuador	61	(2.6)	12.1y	71	(3.3)	14.2y	**	(0.2)	**	(0.4)
India	41	(2.9)	10.0y	47	(3.0)	11.4y	**	**	**	**

Source: Demographic Yearbook 1996, 1998, 2000, United Nations Department of Economic and Social Affairs, New York. Percent surviving based upon data 1992–96 India, 1994, UK and Ecuador, 1999 Sweden. Expectation of life based upon data from 1992 to 96 India, 1995 Ecuador, 1998 USA, 1999 Japan, Sweden, and UK (** indicates data were not available for this item) [12–14]

Long-term evolutionary trends pattern senescent related somatic, physiological, and functional declines. The pace of declines varies between individuals and groups due to genomic attributes, environmental settings, sociocultural conditions, physiological resilience, and individual experiences [8–10]. Multiple evolved trade-offs and compromises in our skeletal, muscular, physiological, and neurological systems increasingly alter human bipedality locomotion as we age, a clear indicator of declining function. With age, our skeletal structures experience breaks, degenerative joint disease, torn ligaments, crushed menisci, collapsed and crushed vertebrae, osteoporosis, and other degenerative conditions. These limit mobility and contribute to increased skeletal frailty. Species wide patterns of muscle and bone loss, changing anthropometric dimensions, physiological, sensory, and functional declines, losses of abilities and reduced mobility, provide useful data for those designing senior living environments to improve accessibility and accommodate frail residents.

Across built environments of the 20th - twenty-first century, increased survival (Table 1) reflects social, medical, and technological advances in providing sufficient food, shelter, sanitary systems, public health, and biomedicine to about 7.7 billion people worldwide. Although common, late-life survival still varies widely across populations, e.g., 41% of men and 47% of women in India survive 70 years, while in Japan 75% of men and 88% of women do (Table 1). Life expectancy at age 70 is relatively consistent across populations (10.0 to 13.5 years men; 11.4 to 17.7 years women), as it is at 85 years (Table 1). Such survival increases proportions of seniors within populations (Table 2), and thereby the number at risk for degenerative conditions, disability, dysregulated physiology, and frailty [1, 15–18]. With age, our abilities to interact with natural and built environments decline, reflecting declining strength, mobility, perceptions, and increasing frailty. Such limitations may also reflect failures of human-environment interfaces to accommodate frail seniors when designed for healthy and hale phenotypes. In general, congregate living/residential communities, private homes, their artifacts (e.g., furnishings, fixtures, décor, tools, implements), interiors (e.g., hallways, kitchens, baths, private rooms), and apparatuses (e.g., baths, beds, lighting, plumbing, showers, windows) were not designed to accommodate fully functional and physical declines among frail seniors.

**Table 2:** Life expectancy at birth 1960 and 2016 and percent increase in population aged over 50 years in selected nations

Nation	1960	2019	% Increase
Japan	68	84	23.5
UK	71	81	14.1
Sweden	73	83	13.7
USA	70	79	12.9
Ecuador	53	77	45.3
India	41	70	70.7
China	44	77	75.0
Poland	68	78	14.7

2019 Data: The World Bank 2018, Life Expectancy at birth accessed 12 November 2018 [19].. https://data.worldbank.org/indicator/SP.DYN.LE00.IN. 2016 Data [1]

Based on human ecological theory and research across physiological anthropology indicating environments become more influential on human function and physical abilities with age, this conceptual review explores age-related declines in human abilities and increasing frailty [1, 3, 5, 20–22]. Herein, I illustrate how declining physical capabilities and function underlie increasing systemic frailty with age that limit seniors’ abilities to interact with their natural and built environments. My goal for documenting late-life frailty and human-environment interactions is to present data in support of designing functional housing for accommodating frail seniors, while enhancing their well-being and quality of life (QOL).

Although paced differently across individuals, everyone’s cognitive and physical abilities, physiological function, resilience, and strength decline with age. Similarly, all who achieve late life eventually experience frailty, leaving them dependent on residential and environmental settings for accommodating their limitations. Given all seniors experience unique socioenvironmental stressors over their lives, sources of age-related variation are complex. However, in aggregate late-life somatic declines and frailty are predictable, patterned, and trackable. Understanding this late-life frailty potentially aids in developing built environments to better accommodate not only frail seniors but all who require such. This requires empirical and observational data on how seniors’ capabilities change, and frailty increases with age. Attention to functional and physiological changes and increasing frailty of seniors during late life while designing and redesigning residential settings is likely to aid in supporting their mobility, activity level, health, and QOL, while also possibly limiting additional frailty [21, 23–25].

Major demographic changes shaping populations over the twentieth century and into the 21st were declining birth rates and increasing survival rates. Improved survival led to worldwide increases in septuagenarians, octogenarians, and nonagenarians and 50% survival to 85 years in some populations (Table 1). Concurrently, frailty and disability shifted to older ages. Changing demographic profiles, increasing lifespans and frailty challenge governments and designers to provide appropriate housing options for seniors. Further, they challenge physiological anthropologists and other researchers to provide an empirical base for developing housing and environments that accommodate observed somatic losses, declining capabilities, and increasing frailty of older citizens [22, 24, 26, 27].

### Aging and the aged

Following reproductive adulthood (~ 55 years), mortality increases exponentially as lifelong stressor exposures, deteriorating molecular, physiological, and cognitive processes differentially affect individuals [1]. Current survival patterns of increasing survival suggest a late-life LH phase is now being expressed by humans in built environments worldwide [1, 5, 6]. A LH phase characterized by chronic, progressive, and deleterious alterations in body habitus, physiological function, bone and skeletal structures, strength, physical capabilities, sensory perceptions, endurance, and resilience, i.e., senescent biology [5, 6, 28]. Such alterations decrease physical and physiological capabilities, function, and physical activity, contribute to frailty, hamper mobility, and alter how individuals use space [29]. Before using environmental designs to accommodate seniors diminishing physical capabilities and functional needs, it is necessary to first document and understand their patterns of age-related somatic and sensory losses [22].

## Senescent biology

Following reproductive maturation, humans generally maintain their maximum capacities through an adult period of reproductive effort, approximately ages 20–50 years [1, 5, 6, 11, 28, 30, 31]. Thereafter, species-specific LH traits interacting with individual genomes play out within a context of ancestral backgrounds, socioenvironmental experiences, and the availability and security of built environments, jointly structuring individual reactivity, endurance, resilience, and, ultimately, frailty. Following a period of maximum somatic strength and function during adulthood, use and disuse, stressor-related damage, wanning endurance and resilience, and cellular senescence consistently promote a frailer senescent phenotype [1, 5, 6, 31]. As early as our fourth decade, we show reduced strength secondary to incipient muscle and bone loss [18, 32–34]. Concurrently, physical decrements, along with cognitive and sensory losses alter our perceptions of the world and others. This leaves seniors more dependent on their built environments for support than younger cohorts as frailty and increasingly limited abilities alter their human-environment interactions.

### Changing phenotypes

As we age, our phenotypes differ significantly from earlier optima [1, 28, 31, 35]. Reduced muscularity, strength, statue, and sensory perceptions are obvious. Less obvious are altered cells and organs, losses of active mitochondria, and slowed neurological and physiological responses. Muscle and bone losses alter body composition and habitus (e.g., percent muscle, bone, and fat, weight, skinfolds, fat distribution), while reshaping our somas (e.g., height, sitting height, waist/hip ratio) and reducing functionality. Strength, endurance, and sensory perceptions (e.g., visual, auditory, tactile) decline, while physiological parameters (e.g., blood pressure, glycaemia, hormone profiles, energy use) are reset to accommodate current needs and limited capabilities. Jointly, these diverse processes underlie physical instability, altered postures and gaits, reduced balance, muscular-skeletal function and mobility.

Longitudinally assessed anthropometric, biometric, and physiological trait distributions identify ranges and changes in quantitative phenotypes. For example, in both longitudinal and cross-sectional analyses, height, weight, strength, and even BMI tend to decrease as we age (Tables 3, 4, 5). Unfortunately, due to small numbers surviving, final age groups for such distributions frequently are truncated at 65+ or 70+ years. However, phenotypic variation continues accumulating well into our 8th, 9th^,^ and latter decades. To design spaces capable of accommodating frail seniors while fitting their ergonomic needs requires population-specific data on variability in quantitative and qualitative phenotypes as these change through late-life (Tables 3 and 4 illustrate height, weight, strength variability at older ages).

**Table 3:** Average height (cm), weight (Kg), and body mass indices (BMI) of adults by age group in the United States 2015-2016^a^ and Japan 2016^b,c^

	Men			Women		
Age	Height	Weight	BMI	Height	Weight	BMI
**United States**						
20–39	176.1	89.3	28.7	162.7	76.0	28.7
40–59	175.8	91.1	29.4	162.1	80.0	30.4
60+	173.4	88.3	29.3	159.3	75.5	29.8
All	175.4	89.8	29.1	175.4	77.4	29.6
**Japan**						
30–34	172.7	67.6	22.6	157.5	51.7	20.8
50–54	167.6	68.5	23.6	157.5	53.1	21.4
65–69	165.1	63.5	23.3	152.4	52.2	22.5
75–79	162.6	61.2	23.2	149.9	50.4	22.4

^a^Mean Body Weight, Height, Waist Circumference, and Body Mass Index Among Adults: United States, 1999–2000 Through 2015–2016, Table 1: weight and 3: height [36]. https://www.cdc.gov/nchs/data/nhsr/nhsr122-508.pdf Accessed 10/18/21

^b^Heights and weights of Japanese men by age group [37]

http://nbakki.hatenablog.com/entry/Average_Weight_of_Japanese_2016 and http://nbakki.hatenablog.com/entry/Average_Height_of_Japanese_2016. Accessed 10/18/21. Source: Japan Sports Agency

^c^Average Weight of Japanese, 2016: Ministry of Education, Culture, Sports, Science and Technology [38]

https://nbakki.hatenablog.com/entry/Average_Weight_of_Japanese_2016 Accessed 11/12/21

**Table 4:** Heights, weights, standard deviations, and *p*-values for liner trends in height and weight 1999–2000 to 2015–2016 among adult men and women by age group in the United States^a^

**1999–2000**										
	**Men**					**Women**				
**Age Group**	**Height**		**Weight**		**BMI**	**Height**		**Weight**		**BMI**
20–39	176.2 (0.2)		84.3 (0.9)		27.2	163.2 (0.3)		73.4 (1.0)		27.9
40–69	176.3 (0.4)		88.1 (1.2)		28.3	162.8 (0.4)		76.8 (1.3)		28.9
70+	173.5 (0.3)		85.2 (0.8)		28.3	159.0 (0.2)		71.6 (0.6)		28.3
**2015–2016**										
	**Men**					**Women**				
	**Height**	**p**	**Weight**	**p**	**BMI**	**Height**	**p**	**Weight**	**p**	**BMI**
20–39	176.1 (0.3)	.096	89.3 (1.4)	<.001	28.8	162.7 (0.4)	.407	76.0 (0.8)	<.001	24.2
40–69	175.8 (0.4)	.096	91.1 (0.9)	.032	29.5	162.1 (0.4)	.041	80.0 (1.4)	.026	30.4
70+	173.4 (0.5)	.965	88.3 (0.8)	<.001	29.3	159.3 (0.5)	0.588	75.5 (1.2	<.001	29.7

^a^Mean Body Weight, Height, Waist Circumference, and Body Mass Index Among Adults: United States, 1999–2000 Through 2015–2016, Table 1: weight and 3: height [36].. https://www.cdc.gov/nchs/data/nhsr/nhsr122-508.pdf Accessed 10/18/21

**Table 5:** Averages, 10th and 90th percentiles, and percent declines in average handgrip strength (kg) by age group for men and women in the USA, China, and Mexico (percentiles not available for Mexico)

**United States**								
	**Men**				**Women**			
**Age Group**	**Grip**	**Percentiles**		**Percent**	**Grip**	**Percentiles**		**Percent**
		**10%**	**90%**	**Decline**		**10%**	**90%**	**Decline**
25–29	47.0	33.7	66.2		29.6	20.2	39.7	
60–69	38.4	23.3	52.5	18.3	23.6	11.7	31.2	20.3
80–85	28.1	15.6	38.2	26.8	19.9	14.5	27.0	15.5
**China**								
	**Men**				**Women**			
**Age Group**	**Grip**	**Percentiles**		**Percent**	**Grip**	**Percentiles**		**Percent**
		**10%**	**90%**	**Decline**		**10%**	**90%**	**Decline**
18–29	35.6	28.9	41.7		22.0	15.9	27.6	
60–69	29.5	21.8	38.7	17.1	18.6	13.1	24.0	15.5
80+	20.9	12.5	27.8	29.2	13.6	8.6	18.5	26.9
**Mexico**								
**Age Group**	**Men**		**Percent** **Decline**		**Women**		**Percent** **Decline**	
20–29	44				28			
60–69	38		13.6		22		27.3	
70+	33		13.2		22		0.0	

Data Sources: Mexico [39], United States [40], China [41]

Many quantitative physical (e.g., weight, stature, walking speed) and physiological biomarkers (e.g., blood pressure, fasting glucose, heart rate) vary with age. More extreme phenotypes (e.g., hypertension, hyperglycemia, extreme overweight or underweight) tend to experience earlier mortality. Those closer to their cohort averages, tend to survive into later decades. Consistent associations of such biomarkers, along with other neurological, physiological, somatic, and immunological assessments of dysfunction with mortality support use of biomarker composites as estimates of individual health, frailty, disease, and death [42–48]. Such composites generally include biomarkers of neurological, physiological, somatic, and immunological dysfunction that differ between age groups. Biomarker-outcome (e.g., mortality) associations generally are similar across groups, but not always. One consistent finding, seniors with lower abdominal fat and/or BMI show elevated mortality risk, contrary to observations on younger cohorts [49–51]. In a similar fashion, elevated cholesterol is less closely associated with risk of death at older ages. Such reversals and absences of association may not reflect reduced risk. Rather earlier selective mortality or detrimental physiological alterations elsewhere may differentially influence related risks with increasing age.

### Body habitus and mobility

#### Anthropometric variation

Anthropometric assessments originally were developed to monitor child growth and development and led to standardized growth charts [52]. Later, anthropometry was extended to assessing adult health across populations and lifetime variation in body habitus [53, 54]. Worldwide, humans tend to follow a consistent pattern of growth, development, maturation, and aging [2, 4]. At older ages, we tend toward greater variation in life-long somatic trajectories but generally we are shorter and lighter than our cohort averages at middle age. In the USA and Japan seniors show shorter heights and lower weights than their middle-aged compatriots. They also are shorter than their cohorts during midlife but show a similar BMI (Tables 3 and 4), suggesting slightly shorter phenotypes may have survived longer.

#### Skeletal change

Osteoporosis (loss of bone matrix) is a universal of human aging [55]. As bones become increasingly porous and fragile, strength and mobility are reduced. In addition, damaged cartilage, ligaments, and tendons, along with compressed/fractured bones, arthritic joints, damaged/collapsed intervertebral discs, flat feet, and contractures impair skeletal function, reducing physical activity and contributing to frailty. Stress also enhances glucocorticoid excretion, promoting apoptosis of bone-forming cells [56]. At reproductive maturity, women show less dense and robust bones than do men. They also experience more rapid bone loss over their lives, including post-menopausal osteoporosis [18, 56]. Bone loss with age is further compounded by collagen cross-linking in tendons and ligaments, another contributor to postural and gait alterations limiting mobility. As bone loss proceeds, sitting height declines, while leg length remains relatively stable, leaving seniors with shorter sitting and standing heights. As a result, they may be poorly accommodated by chair backs, seat heights, and facilities designed for taller, more robust, and stronger individuals [57, 58]. By altering agility, balance, and gait, age-related bone loss also contributes to frailty.

Dysregulated bone physiology has somawide detrimental effects. Altered skeletal biology may limit hematopoietic cell output, alter immune function, or reduce systemic oxygen-carrying capacity, compromising overall health and increasing frailty. Consequent to differences in physical activity, nutrition, trauma, and senescent biology, bone loss and skeletal dysfunction occur at variable rates and ages, but ultimately affects all. United States Medicare beneficiaries illustrate the ubiquity of age-related bone loss. In 1999–2005, of recipients aged 65+ years, 25% reported osteoporosis; among those receiving benefits 6–7 years, 42% of women had osteoporosis, 10% of men did [18], suggesting women live with greater frailty than men in later life. This also may reflect selective mortality, as fewer men survive to older ages.

#### Skeletal function

At all ages, appropriate skeletal function and biomechanical stability promote mobility, sociality, physical activity, health, and survival. Skeletal and muscle cells are integral to systemic biology, participating in blood cell production, immune competency, calcium and potassium balance, and mineral storage. As we age, bone microfractures are less fully repaired, leaving bones weaker; collagen glycation contributes to brittle, hard, and less elastic cartilage and compressed intervertebral disks, further limiting activity. Concurrently, cartilage on articular surfaces hardens, becoming brittle and compressed, while damage from wear and tear, accidents, injuries, and fractures accumulate. With cellular senescence aggravating this cycle, integrated systems supporting mobility deteriorate and functional losses lead to low physical activity and greater frailty.

#### Height, weight, BMI

In addition to musculoskeletal changes leading to sitting height reductions with age, secular trends in stature contribute to older cohorts being shorter and lighter than their younger counterparts and themselves during middle-age (Tables 3 and 4). In 2005, Japanese men aged 60–67 were 8 kg lighter than when aged 40–49, and lighter than men aged 40–49 years [59]. Similarly, across eight samples, Annis [57] reported weight gain through the fifth and sixth decades, gradual loss through the eighth decade, and rapid loss thereafter (Tables 1 and 2, p.383). Over recent history, taller adults have experienced higher mortality than shorter; a trend clearer from the1960s through 1980s, than more recently [57, 60]. Among men, height decrease ranged − 1.2 cm to − 9.9 cm, among women − 1.4 to − 6.3 cm, between ages 20–29 to 60–69 years across eight samples [57]. Similarly, long-lived Okinawan men (ages 87–104 years) are shorter and weigh less than nonagenarians and older long-lived men (ages 100–105) residing elsewhere in Japan [61]. Okinawan women show a different pattern, being taller and heavier at ages 87–104 than Japanese women aged 100–105 residing elsewhere in Japan [61]. However, in this comparison Okinawan women are significantly younger than are centenarian Japanese women from elsewhere. Overall, in both USA and Japan, those surviving to later ages are shorter than their middle-aged counterparts (Tables 3 and 4). Shorter height may not associate significantly with older age in all settings. For example, in the Netherlands Cohort Study (120,852 men and women ages 65–70 at baseline, self-reported data), men attaining their 90th birthday showed no significant difference in height compared to those who did not, while women surviving to 90 years were significantly taller than those not [62]. Although appearing contrary to the general trend, in addition to being self-reported, these data do not represent a complete birth cohort, only those who survived to ages 65–70 were included. It is not known if survivors to age 65–70 and age 90 years were shorter or taller than their cohorts were when 30–40 years, the more accurate comparison group. Across populations, a majority of height loss with age is sitting height [57], suggesting height loss reflects spinal alterations and compression, e.g., collapsed vertebrae, osteoporosis, work and activity related injuries, and stressors. Such spinal degeneration impinges on balance and gait and limits mobility, contributing to frailty. Contrary to these historical analyses, in recent decades, across many settings worldwide, taller people are surviving longer, while experiencing less frequent death from heart disease and stroke than shorter [63, 64].

#### Sarcopenia

Muscle loss is directly and independently associated with chronic disease and shorter life spans [65]. With increasing age, sarcopenia affects everyone, with women commonly affected at earlier ages [32]. Sarcopenia reflects lifelong behaviors, occupations, lifestyles, experienced stressors, and innate biology. Given less robust and weaker muscles, along with osteoarthritis, bone loss, compressed/fractured vertebra, and damaged knees, seniors tend toward altered gaits, locomotor instability, greater double support (time balanced on both feet), shuffling, and stooped postures. In addition, knee, leg, and back pain limit agility, physical activity, and mobility, while increasing risks for accidents and falls and compounding frailty.

#### Activity patterns and mobility

Across populations anthropometric dimensions of seniors differ from younger adults, indicating less robust physiques (Tables 3 and 4) [28, 66]. With increasing age, declines in strength, physical, and physiological capabilities limit abilities to accomplish necessary tasks of life (e.g., self-care, mobility). Across species, late life predicts somawide reduced/compromised physiological capabilities: e.g., nerve conduction velocity, hormone titers, blood pressure, stressor responses, diurnal cycles, compared to earlier adult ages. Interactively, these alterations limit physical activity and strength-related tasks, inhibit self-care, and reduce mobility, compounding frailty and producing variable capabilities and activity patterns among seniors. As losses continue, endurance and resilience are compromised, contributing to frailty, and further reducing interpersonal and environmental engagement. Whether residing alone, with a spouse, or in congregate residential settings, seniors with limited mobility experience limited social interactions, increased isolation, less life satisfaction and lower QOL [67].

A major goal of aggregate senior housing designs is providing support for residents’ continuing function, independence, physical activity, and mobility, while enhancing their abilities and accommodating their limitations. For example, walking gaits and mobility aids used by many frail seniors are less compatible with some indoor walking surfaces (e.g., carpets, runners, tiles) and outdoor (e.g., gravel), than they are with some natural surfaces (e.g., wood, grass) or even concrete. However, tile, concrete, and asphalt are compatible with the wheeled carts, chairs, equipment, and sanitation needs common to congregate residential settings. When wet, these increase risks for falls, muscle, and skeletal damage, particularly among mobility impaired residents.

Over recent decades, newly designed and renovated residential senior housing projects have moved toward more accommodating designs. Many have replaced steps with ramps where possible, installed wider elevators, doorways, and hallways, and/or improved resident access to interior and exterior environments. Incorporating access to outdoor areas, gardens, interior/exterior courtyards, internal and exterior walking, wandering, and rolling pathways also supports resident mobility and physical activity, while enhancing their sociality, well-being, and health, and possibly limiting additional frailty [68]. Still, seniors do not share identical phenotypes. Their lived experiences in variable environmental settings produce a range of cognitive and physical phenotypes at older ages. Built environments designed for access by and accommodation for highly variable, frail seniors will improve their mobility, physical activity, and social engagement, while also enhancing accessibility for all age and ability groups. Connecting interior with exterior spaces improves physical activity, while allowing residents to engage with nature, access sunlight and fresh air, and exercise their visual and audio acuity, activities known to improve well-being and reduce stress among seniors.

Physical abilities likely directly influence seniors’ housing choices. Both quantitatively (accelerometer) and qualitatively (self-reports), at ages 60+ years seniors in private homes engage in and report greater physical activity than those in congregate settings [69]. In general, less frail seniors may choose to remain at home, while frailer seniors choose congregate settings. Designing to maintain and improve residents’ well-being and limiting frailty through physical activity, represents a priority for congregate senior residential settings. Given living in private homes requires greater physical activity, promoting similar activity patterns among residential seniors may be a useful design consideration when building environments for seniors. Doing so requires observational and self-report data on activity patterns and social interactions among seniors living in private homes. Such data may spark innovative design concepts for improving physical abilities and reducing frailty among seniors residing in congregate settings. Although design attributes may not reduce frailty to levels seen among independent-living seniors, they may aid in slowing the progress of frailty, while improving mobility, health, and QOL.

### Sensory losses

Beyond somatic and physiological limitations, with age our neurological innervation and signal processing speed and capacity decline, reducing sensory acuity [28, 35, 70, 71]. These alter perceptions, increase response times to stressors, impair health, limit mobility, hinder socialization, and reduce QOL and life satisfaction. Sensory losses also inhibit abilities to perceive our environments, artifacts therein, food aromas and tastes, and sense dangerous odors.

#### Sight

Even without obvious pathology, visual acuity declines with age [28]. Secondary to stressor exposures and senescent biology, lenses become opaque, cataracts form, macular degeneration progresses, and pupils dilate slower reducing accommodation, while our abilities to perceive the visual spectrum and differentiate shapes, colors, and textures decline [24, 63, 64]. Multiple environmental (e.g., dust, pollen, ultraviolet radiation, wind, infectious and parasitic organisms), sociocultural (e.g., hazardous occupational exposures, violence, diets, availability of medical care and surgery), and personal factors (e.g., exposure to ultraviolet radiation, accidents, toxins, self-care, behaviors) influence sight among seniors. Across individuals, properties of their visual cortex and eyes respond differentially to variable lighting sources, natural and artificial light, its intensity, brightness, and color, significantly influencing visual acuity and perception. Multiple aspects of built environments, sizes, colors and shapes of spaces, time of day and structural features, furnishings, and users’ activities influence individual abilities to see.

Lighting, along with furnishings and color, set the mood for a space, influencing perceptions, emotions, and behavior even in identical spaces [72–74]. By accentuating properties and functions of a space, lighting systems identify its current use. Within and outside residences, lighting provides navigational and location cues, accommodating visually and directionally impaired residents. Variable lighting types also influence perceptions of color and texture. In congregate settings, inappropriate lighting may alter the look of food making it appear off-colored, oddly textured, or less than fresh, thereby possibly influencing food choices [75], and limiting caloric and nutritional intakes.

Many natural and artificial lighting options are available to fulfill interior and exterior illumination needs for safety, mobility, and activities whether in congregate or private housing. For task-specific spaces: e.g., completing activities of daily living (ADLs) and instrumental activities of daily living (IADLs), bathing, self-care, food preparation/consumption, entertainment, physical activity, and sleep; multiple possibilities are available. Importantly, within congregate communities, lighting type identifies specific locations, pathways to them, stairwells, and elevators. Variable lighting also demarks personal, community, interior, and exterior areas. As individuals vary in their perceptions of comfort, brightness (lumens) and color (light appearance) of lighting within spaces should be adjustable to user preferences for that settings. Determining appropriate illumination depends on knowing intended purpose of the space, types of activities, expected occupants, and proposed furnishings. For senior residences, one suggestion is general lighting within the community be high lumen (1600+) with a relatively cool color temperature (3000–4000) [76]. This should provide appropriate illumination in public areas, e.g., dining, food service, lounging, reception/visiting, while accommodating all users, including visually impaired [76]. Within less public and more private areas, a variety of lighting patterns and illumination levels may be appropriate. Remote controlled adjustable lighting is a basic enhancement for private and communal spaces. This allows each occupant to achieve their current personal preferences, while better accommodating visual impairments, mobility limitations, and frailty, and supporting autonomy. Throughout congregate communities, ceiling, wall, floor, table, and corner lighting provide both ambient and guide lighting. Accent lighting enhances design features, drawing attention to specific items and visually augmenting characteristics of a space. Conversely, task-specific lighting provides sufficient brightness and color temperature to enhance completion of specific activities, e.g., bathing, cooking, reading, writing, TV-watching. To enhance visual acuity, within private settings, task-specific lighting should be adjustable to individual residents’ physical needs and capabilities. Type and quality of lighting exposure also may influence personal health. For example, Yasukouchi et al. [77] compared participant responses under either incandescent or Rayleigh scattering lighting. With Rayleigh lighting, participants reported feeling more natural, while also showing lower blood pressure and cortisol arousal, and higher nocturnal melatonin secretion. In senior housing, Rayleigh skylights may be useful for improving affect, while also reducing physiological risk factors. Conversely, excessive overhead lighting may convert a space from appearing warm and inviting, to feeling artificial and less than homelike. In senior housing, lighting designs combining multiple light sources, overhead, table, floor, and accent lighting and remote control provide more balanced lighting schemes.

Before designing lighting and illumination patterns for new or existing residential communities, participation observation, including interviews with and reports from current residents, staff, visitors, and administrators of existing communities, is necessary to determine specific lighting goals and appropriate designs to achieve them. An important final design step is post-occupancy follow-up with residents and stakeholders to determine how designs fit needs, thereby contributing to a growing database on accommodating lighting and other design items to frail and hale seniors [77, 78].

#### Hearing

As does sight, auditory acuity fades with age, altering our perceptions and awareness of others, social cues, environmental risks, and stressors. Faded hearing also slows response time to alarms, announcements, and traffic and leads to loud voices, televisions, radios, and phones. With age, reduced sensitivity to audio frequencies, tinnitus, and other issues interferes with auditory comprehension, and hearing aids become common. Many existing homes and congregate residences were designed with less attention to accommodating late-life auditory impairments than today [79]. Exterior designs have a long history of noise abatement, e.g., shrubs, trees, logs, fences, stone walls, embankments, while interior noise abatement has been more limited in application [79]. Modern interior designs incorporate multiple sound-retarding features, drapes, acoustic panels, spongy fiber, wool, wood, cement, and laminated walls, floor coverings, and furnishing. Additionally, new construction is being designed with multiple structural factors to limit sound propagation, e.g., decoupling structural elements, internal damping, insulation, and air spaces [80]. As designers increasingly address interior sound abatement, this list of sound reducing possibilities is expanding [79]. For interior spaces, barriers, e.g., walls, half walls, live and artificial plantwalls, and other dividers, along with sound-retarding furnishings, decorative elements, spatial layouts, and smaller spaces, reduce sound propagation. Over the late twentieth century, a major design trend for private and congregate housing was great rooms and multipurpose areas wherein activities, e.g., dining, entertainment, TV watching, were combined into one space. Designed to unify family and group activities [81], large open spaces may poorly accommodate frail seniors, or those with limited hearing. Research also suggests interior designs reflecting seniors’ past homes, with traditional-sized spaces similar to those wherein their lifelong social interactions with family and friends occurred, furnished with homelike and natural products (e.g., wood, stone, bamboo) and plants, provide more relaxing and natural acoustical settings [72, 73, 82, 83]. Such settings may better accommodate seniors’ auditory and visual needs, while supporting their communication abilities and providing feelings of familiarity and home.

#### Taste and smell

As taste buds and olfactory neurons decline in number, cease function, alter internally, senesce, or do not respond due to dysfunction elsewhere with age, acuity of taste (gustation) and smell (olfaction) decline. Of our five tastes (i.e., salty, sweet, bitter, sour, umami), salty and sweet tend to decline most with age. Subsequent reduced taste sensitivity may predispose to overuse of salt and sugar as condiments, thereby contributing to risks for hypertension, diabetes, and other NCD. Taste bud losses and aroma-sensing neuron declines may reduce intakes of necessary dietary components, e.g., vitamins, proteins, carbohydrates, fiber, fats, increasing risks for malnutrition, undernutrition, and associated health problems. As such, altered taste and olfactory sensitivity may lead to reduced dietary breath and nutritional insufficiencies. Nutritionally complete diets are basic to good nutrition for all, but particularly seniors. Undernutrition and malnutrition increase seniors’ risks for frailty, morbidity, and mortality [84].

How prepared food appears, smells, tastes, and its mouthfeel (tactile perception) influence food choice and thereby dietary adequacy. Olfactory losses may alter aromas and tastes of foods. Other than proper ventilation and sanitation facilities, few design features are available to attenuate dietary issues related to food taste and aroma. Among those with visual problems, food may appear unnatural in color or texture, making it less appealing to eat and difficult to distinguish among different food types. Olfaction determines how food aromas are cognitively interpreted, and related cognitive losses may reduce or alter food intake, thereby influencing health. Besides limiting nutritional intakes, reduced olfactory sensitivity is a physiological risk, Rawson et al. [85] reported 45% of seniors could not detect the smell of natural gas in their environment. As mentioned earlier, lighting schemes may aid in enhancing food appearance and color, thereby improving consumption. At all ages, nutritionally complete diets are basic to good nutrition. In any setting, appropriately designed kitchens and serving areas with sufficient space allow appropriate, appealing, and tasteful foods to be provided.

#### Tactile

Tactile sensitivity (touch), kinesthetic perceptions, and sensitivity of skin to temperature fluctuations decline with age. Responsiveness of proprioceptors along with output from fibroblasts (e.g., collagen, extracellular matrix) and dermal cell numbers decline, while underlying skin layers atrophy, dermal layers shrink, thin, and become less pliable. Losses of subcutaneous fat and dermal tissues, cross-linking of collagen fibers, and decreased innervation reduce sensitivity to external temperature changes, while increasing response time to core temperature changes. During heat waves, poor response to overheating contributes to higher mortality among older and immature age groups than among reproductive age individuals (see for example: China [86], Europe [87]. Skin, a protective barrier from the environment, thins with age increasing susceptibility to injury and infection and reducing environmental awareness and perception. In association with visual losses, age-related declines in tactile sensitivity reduce our abilities to negotiate natural and built environments and mobility.

Variability in genotypes, environments, growth and development, and investments in somatic maintenance contribute interactively to sensory and perceptual losses. In turn, sensory losses may contribute to decreased dietary breadth, poor nutrient intakes, muscle loss, reduced physical activity, slowed response to stressors, decreased mobility, and frailty. All who survive to late life experience changes in their sensory perceptions. By understanding how our sensory abilities decline with age, those developing residential settings have models and evidence to aid them in supplementing, accommodating, and overcoming limitations imposed by senescent biology through the design of our built environments.

### Male-female differences

One additional, oft under-considered fact when designing residential settings for seniors, whether frail or not, is their demography. Specifically, more women survive to late life, outliving men across all national populations [88–90]. In Japan, women are twice as likely to survive to age 85, in the UK 85%, in the USA 75%, and in Sweden 73% more likely (Table 1). Throughout life, females tend to express greater somatic resilience to environmental stressors [91]. Current longer life spans among women reflect evolutionary pressures on somatic, hormonal, and physiological resilience related to pregnancy, birth, lactation, and childcare across our genus. Still, during their longer lives, women report more illnesses and experience more osteoporosis, sarcopenia, and disability than same-aged men, who experience higher mortality at all ages. A pattern exhibited across all human and most mammals and described as a male-female morbidity-mortality paradox [88–90, 92–94]. For now, and the future, most seniors in congregate residential and long-term care settings are and will be women. Those currently in congregate residential settings provide a model for designing accessible and accommodating environments for seniors, particularly for frail elders. Late life and frailty are more prevalent among women than men. As frailty mostly affects senior women, they are appropriate model residents for determining how environments improve and limit access by and accommodations for frail seniors. Improving accessibility of interior and exterior building designs for senior women, will equally benefit senior men and most user groups.

## Frailty

Over human life, cellular senescence, stressor exposures/responses, and physiological dysregulation limit somatic function/abilities leading to deleterious phenotypic alterations as we age. To appropriately target clinical care to increasingly frail seniors, multiple evidence-based indices of physiological, neurological, and functional capabilities are available. Among the familiar are Activities of Daily Living (ADL) [43], Instrumental Activities of Daily Living (IADLs) [95], biological age [45, 96, 97], allostatic load [48, 98, 99], frailty [9, 32–34, 100], deficit index [44], and Framingham Score [101]. Theoretically, these indices assess preclinical/clinical somatic and physiological dysfunction. Practically, they associate significantly with current and future health, disability, morbidity, and mortality [18, 32–34, 44–46, 100–106]. Each integrates multiple assessments of inherent and individual alterations in phenotypes; physiology, function, well-being, and influences of senescent biology, into a single estimate. For example, frailty is described as a specific clinical phenotype commonly assessed by multiple biomarkers of strength, endurance, mobility, and physical activity/ability [18, 32–34, 104–106]. In the living, frailty primarily reflects loss of muscle cells (sarcopenia) and skeletal matrix (osteopenia, osteoporosis), leading to reduced strength, endurance, and abilities [32–34]. Multiple frailty indices, based on assessments of current somatic and physiological function, capabilities, and limitations have been proposed [32, 100, 102, 107, 108], some including as few as 5 assessments [32–34], others extending to 50+ assessments across multiple domains [100, 102, 107, 108]. As a somawide phenotype, frailty reflects reduced strength, resilience, capabilities, and endurance, increases during late life, and underlies incipient limits to mobility, while increasing more rapidly among children of short-lived, than long-lived, parents [18].

Across modern populations, a major contributor to frailty is sarcopenia. For example, grip strength assesses muscle atrophy/loss and documents increasing muscular frailty with age (Table 5) [109, 110]. Among United States adults, grip strength declines through late life (men 40% - women 33%), similar to Chinese men 41% and women 38%, although the former begin adulthood with lower averages (Table 5). Molecular variation, physiological resilience, lifestyles, and availability of medical intervention influence individual frailty. For example, in a sample of 200,000 Europeans ages 60–70 years, sarcopenia and frailty associated significantly with alleles influencing insulin and obesity [111]. All survivors to late life eventually experience frailty, suggesting frail phenotypes provide living models for designing accommodating and accessible environments for all who achieve late life.

Limiting negative impacts of frailty on older adults provides a specific goal for designs of built environments to house them. Frail seniors provide the evidence-based framework necessary for designing built environments that enable and accommodate frail phenotypes of all ages. Spaces designed to limit frailty progression, encourage physical activity and social interactions, and improve users’ abilities to complete daily activities will help limit frailty and benefit all user groups. Physical activity, exercise, walking, and strength training all help limit frailty and maintain mobility. Similarly, positive social interactions are associated with physical and mental health, fewer functional limitations, and lower frailty. Innovatively designed environments, interior furnishings, and appliances directly addressing frailty among seniors will encourage physical and social activity, improve access for while better accommodating frail seniors, and aid them in maintaining their abilities while slowing their frailty trajectory.

### Frailty and accessibility

Frailty limits our physical capabilities, thereby inhibiting abilities to access and use private and public environments, built spaces, and manufactured objects. Accommodating environments and products to specific-needs and user groups is a universal design (UD) principal. Others include accessibility, equitability, and flexibility in use; simple and intuitive to use items; perceptible information on proper use; high tolerance for user error; usable with low effort; and available in sizes and spaces appropriate for all users [112]. Whether in private homes, congregate residences, or long-term care, frail individuals of all ages are a specific-needs user group. Documenting frail seniors’ interactions with built environments provides an evidentiary base to address accessibility and accommodations for all user groups.

Originally, senior congregate residential housing was viewed as an extension of medical care, leading to designs reminiscent of hospitals, as health care for mental and physical ills were primary concerns. Viewed as healthcare sites, through the 1970s designs for senior housing communities reflected this medical model. Designs for maintaining and improving lost capabilities, while accommodating age-related declines and impairments were seldom emphasized. Research among seniors in congregate housing during the late twentieth century led to new views of senior congregate housing as living environments wherein design innovations accommodating long-lived and frail residents could improve their QOL, mental health, well-being, and life spans. As increasing frailty is an inevitable correlate of increasing age, accessible designs supporting seniors must keep pace. Within many previously built congregate settings, neither exterior nor interior designs, private or public areas, room arrangements, kitchens, bathrooms, lighting systems, nor furniture and furnishings directly addressed the reduced strength, heights, perceptual and sensory acuities that limit seniors’ abilities to interact with their natural and built environments. Such limitations include, most reproductive-age adults being able to open a can of fruit, pressurized jar, or childproof medicine container and raise a 10 kg weight over their heads with little effort, while many over age 75+ fail to accomplish these tasks. Those surviving eight-plus decades are members of a highly variable and unique group. Designing built environments to maintain and improve their limited mobility, physical capabilities, and frailty, while anticipating their increased frailty will best serve their and others’ needs.

### Design considerations

Across populations, although taller people may tend to survive longer today [63, 64], most people surviving to late life tend toward more average than extremes of body habitus. Worldwide, survivors to late life are neither the strongest, heaviest, nor tallest representatives of their cohorts when at reproductive ages. Rather, losses of muscle, bone, and height limit seniors’ strength, robusticity, and flexibility. Further, as age increases, altered gaits, postures, and other physical limitations become more frequent. Most built environments, including furnishings and manufactured items therein, e.g., baths, beds, chairs, lamps, tools, sofas, and utensils, were designed to house relatively able-bodied physiques. These may not accommodate ergonomic needs or anthropometric characteristics of frail seniors.

A major theme within physiological anthropology is documenting how interacting biological, environmental, and sociocultural factors, shape lifelong human variation [97]. Designing built environments and products accessible and accommodating for frail seniors is an ergonomic problem. Given their overlapping interests, collaborative research among designers and physiological anthropologists likely will produce ergonomic insights on current and proposed designs for accommodating built environments to all users, particularly seniors. One example, age-related postural alterations may influence seating choices of seniors. When offered varying height chairs, a self-selected sample of seniors choose heights below standard chair heights, self-reporting better accommodation [58]. One suggestion, seniors prefer lower seat heights for easier access, perhaps due to short stature, limited strength, and/or physical limitations of taller seats. This preference may extend to sofas, beds, and other furniture. Prior to imagining, developing, and initiating environmental designs to accommodate seniors, directed research to attain empirical data, hypothesis testing, participant observation, and user interviews are needed. This aids in establishing an evidentiary base for developing design recommendations for improving accessibility and accommodation of end users. Participant observation and user interviews establish seniors perceived and desired accessibility needs in built environments and help anticipate future needs. At the same time, qualitative and quantitative data help establish desirable attributes for room and furniture sizes, lighting schemes, and dimensions and heights for seating surfaces. Interviews with residents and participation reveal elements within built environment users view as limiting their comfort and accessibility. Spaces, furniture, furnishings, décor, and household artifacts designed to accommodate frail seniors cannot require able-bodied physiques or strength and must be easy and flexible to use. Making frail seniors a blueprint for designs to accommodate all user groups.

### Enhancing lives through design

Many design features, indoor and outdoor courtyards, gardens, and open spaces provide visual and auditory stimuli for, enhance moods of, and allow residents to exercise their physical and sensory abilities [24, 67–69, 83, 113, 114]. However, within many settings, residents using canes, rollers, and mobilized chairs share hallways with caregivers, staff, and equipment. Wider interior corridors, intersections, and doorways better accommodate the mobility enhancing walkers, rollers, and wheelchairs frequent among seniors. Wide short hallways, a frequent component of cluster-housing designs, reduce congestion and may improve resident and staff satisfaction. Circular and cluster housing designs locate private resident rooms in small groupings, along short corridors, with personal room doors opening onto shared “home-sized” public spaces (e.g., lounging, TV-viewing, dining, activity, courtyards). In this arrangement, staff and food preparation areas may be near residential areas; while entrance, reception, and delivery areas may be located away from resident rooms and activities [20]. Cluster designs reduce congestion, limit resident and staff exposures to extraneous distractions, and address privacy issues in personal and daily activities [20].

Today, common congregate care designs include multiple amenities, indoor walking spaces, bird aviaries, sunrooms, fishbowls, along with outdoor porches, courtyards, and walkways. Such additions allow persons with limited abilities or frailty access to outdoor spaces and exterior views, providing sensory stimulation, interactions with nature, and opportunities to exercise distance vision. Although, designing enabling environments for frail seniors is challenging [115], ecological, household, and neighborhood models provide theoretical and applied approaches for developing next-generation care, built environments supportive of frail seniors [20, 69, 115–117].

A seemingly endless array of furnishings, furniture, wallcoverings, textures, accessories, and lighting systems are available for built interiors. For seniors, the simple approach among these many options may be incorporating natural and traditional elements reflecting those they have known throughout their lives into their living spaces. Such additions address seniors’ societal sensibilities and likely will enhance their subjective well-being. For example, Tsunetsugu and colleagues [72, 73] observed “traditional” furnishings, specifically wood panels and furniture, live green plants, and alternative lighting patterns, were judged more pleasing and inviting by Japanese elders, than were spaces furnished with modern office furniture, lighting styles, and lacking natural accessories. These and other data suggest natural and traditional features enhance seniors’ well-being by reflecting feelings of home [73, 83, 84]. Complementing this research, among school children, replacing plastic and metal furnishings with wood and natural products was associated with fewer missed days for illness and better academic performance [82]. Among seniors, furnishings, colors, types of textiles, floor and wall coverings (e.g., wood, cloth, tile, paint), illumination, brightness and color of lighting, along with natural and unnatural products and sounds within built spaces, influence mood, perceptions of comfort and calmness. By modulating such attributes, designers may find ways to better accommodate seniors by enhancing their comfort, likely also improving their well-being.

As described earlier, during the late twentieth century, more homelike designs were incorporated into senior residential communities worldwide. Currently, trends toward decentralized, modular designs continue, with short hallways leading to clusters of individual rooms radiating off a central core and opening onto common activity areas, e.g., dining, recreation, courtyards, seating, with access to interior aviaries, exterior views, and patios [20, 24, 69, 115–117]. Such cluster housing designs allow isolation of reception, nursing, and housekeeping activities within a central core, while private rooms located within smaller modules are accessed by short radiating hallways. Therein living and common areas are centralized while individual rooms radiate from this central core. Cluster design reduces community congestion overall, provides an accessible and accommodating environment for cognitively and sensory-impaired, confused, and frail residents, limits extraneous noise in residential areas, and apparently promotes greater social interaction among residents and staff.

## Discussion

Our ancestors of 30–40,000 years ago were lucky to survive 40 years [121]. Today, you are unlucky if you do not survive 70 years. This extended survival reflects evolutionary responses to environmental stressors our recent and remote ancestors encountered while developing and elaborating humankind’s social and cultural structures [1, 3–6]. Jointly, these enabled them to maintain biocultural settings in which to reproduce and fledge offspring, while retaining sufficient somatic and physiological capacity to eventually survive ten-plus decades [1, 3]. The earliest built structures sheltered, protected, and accommodated needs of local social groups, perhaps as small as nuclear and extended families. In modern settings, built environments are a constant of life housing us all, structuring not only our physical environments, but our social and cultural perceptions. For seniors, particularly the frail, constructed environments often are their world. Thus, they should be designed to enhance their abilities and preserve their somatic reserves while accommodating and limiting their frailty.

With age, we all will develop frailer phenotypes. These hamper our abilities to interact with natural and built environments and make some design attributes in private and public spaces sources of limitation. Designing built spaces supportive of well-being, physical abilities, and accommodating of limitations among frail users provides opportunities for extending health spans among seniors. Frailty indices directly assess such individual capabilities and limitations. Documenting the range of frailty among seniors provides evidence to aid development of supportive designs for improving frail residents’ experiences in senior housing. Designs that provide a common denominator for improving accessibility, inclusivity, and universal usability within built environments, including their furnishings, for all ages and ability levels.

Frailty is a well-documented clinical, epidemiological, and gerontological model and measurable phenotype of age-related functional declines [26, 32–34, 100, 102]. It also is an emerging model in long-term care [118, 119]. Although most common among seniors, frailty also affects the young and middle-aged, suggesting environments and artifacts designed to accommodate frail seniors may accommodate most user groups. For example, within residential settings, natural furnishings, wood products, live plants and organisms, natural and subdued lighting may enhance seniors’ perceptions of comfort and well-being [72, 73, 83]. Similarly, seniors may find furnishings, artifacts, and space attributes (e.g., size, color) similar to those experienced over their lives more relaxing, comforting, and supportive of their QOL than more sterile hospital-like settings. The same is likely true for most groups whom the principles of universal design address.

## Conclusions

This review addresses accessibility and accommodation for frail seniors in congregate residential settings. Most elders prefer to remain in private homes as they age while accommodating their increasing frailty and decreasing mobility by modifying their homes. For seniors, residing in homes apparently requires greater physical abilities, as such individuals tend to engage in more physical activity, while showing and self-reporting better health and well-being than those in congregate housing [116, 119, 120]. Wherever they reside, seniors continually interact with and depend on their built environments and assistive technologies to maintain independence. Across current sociocultural settings, at all ages, built environments are necessary to accomplish life’s basic tasks, e.g., maintaining shelter, transportation, acquiring food, attending medical appointments, and more so among frail seniors. Designs for public, private, interior, and exterior environments in which we reside either enhance or limit our functional abilities, activity patterns, well-being, and QOL. Their greatest impacts fall on frail and older persons. Additional research-based evidence and a database on needs, abilities, and frailty trajectories among frail seniors will aid those designing built environments and housing to reduce impacts of frailty on their activities and lives.

## Data Availability

not applicable.

## Abbreviations

ADLs: Activities of daily living
BMI: Body mass index
IADLs: Instrumental activities of daily living
LH: Life history
NCD: Non-communicable disease
QOL: Quality of life
RC: Reserve capacity
UD: Universal design
